# Bone grafting via reamer-irrigator-aspirator for nonunion of open Gustilo-Anderson type III tibial fractures treated with multiplanar external fixator

**DOI:** 10.1051/sicotj/2017002

**Published:** 2017-04-07

**Authors:** Nicholas Kusnezov, Gautham Prabhakar, Matthew Dallo, Ahmed M. Thabet, Amr A. Abdelgawad

**Affiliations:** 1 Department of Orthopaedic Surgery and Rehabilitation, Texas Tech University Health Sciences Center El Paso TX 79905 USA

**Keywords:** Tibial nonunion, Open tibial fracture, Reamer-irrigator-aspirator, External fixator

## Abstract

*Introduction*: The purpose of this investigation was to evaluate the outcomes following reamer-irrigator-aspirator (RIA) autogenous bone grafting (ABG) of high-grade open tibia fracture nonunions stabilized via multiplanar external fixation.

*Methods*: We retrospectively reviewed all patients with Gustilo-Anderson type III open tibia fractures treated with multiplanar external fixation and who underwent RIA ABG for nonunion at our institutional Level 1 Trauma Center between 2008 and 2015. All patients between 15 and 65 years of age with a minimum of six-month follow-up were included. The primary outcomes of interest were achievement of union, time to union, and incidence of revision surgery. Complications and all-cause reoperation were recorded as secondary endpoints.

*Results*: Fifteen patients met the inclusion criteria with a mean age of 41.1 ± 14.0 years. RIA ABG was harvested from the femur in all cases, with a mean volume of 34 ± 15 mL. At an average follow-up of 13.3 ± 6.8 months, all patients achieved union, including two who required repeat RIA ABG. One patient experienced a femoral shaft fracture four months following RIA that required intramedullary fixation. The average time to union was 6.0 ± 6.3 months. Twelve patients (80%) went on to union within six months and 13 (86.7%) within one year. Five patients experienced a total of six post-operative complications including three deep infections, one refracture through the nonunion site, and one gradual varus deformity. Two patients in this series required a subsequent RIA autografting procedure secondary to persistent nonunion despite initial RIA.

*Conclusion*: We found that RIA ABG offered a reliable solution to nonunion of Gustilo-Anderson type III open tibial fractures treated with multiplanar external fixation, circumventing the need to change the method of fixation.

## Introduction

Tibial nonunion following severe open tibial fractures (modified Gustilo-Anderson classification type III) poses a difficult problem. Definitive treatment with multiplanar external fixation offers a more versatile platform to manage these injuries, as opposed to monoplanar external fixation or internal fixation, permitting deformity correction and compression of the fracture [1]. These are very complex injuries and frequently develop nonunion related to the significant energy imparted during the trauma with resulting disturbance of the local blood supply, poor soft tissue envelope, and potential bone loss [2, 3]. Autogenous bone grafting (ABG) in conjunction with circular fixation may provide an alternative management strategy for nonunion.

Nonunion debridement combined with ABG and bony stability with external fixation is the standard treatment. ABG may be harvested from a variety of sites. As opposed to traditional techniques, the reamer-irrigator-aspirator (RIA) system has more recently been shown to offer an excellent alternative, providing a more minimally invasive technique by which to harvest large volumes of high-quality autograft [4–7]. In the setting of long-bone fractures with small segmental defects, union has been reported in up to 90% of cases [5–9]. However, there are a limited number of series exploring the success of RIA for nonunion following circular fixator application for open tibial fractures [1, 10].

This study presents a consecutive series of open Gustilo-Anderson type III tibial fractures that developed nonunion and were treated with RIA ABG for nonunion following treatment with multiplanar external fixation. The study hypothesized that RIA ABG would provide a reliably high union rate comparable to the existing literature without the need for exchange fixation method.

## Methods

This is a retrospective study approved by the Institutional Review Board. The cohort included all consecutive patients who were treated with RIA ABG for nonunion following multiplanar external fixator application for open Gustilo-Anderson type III tibial fractures between 2008 and 2015 at our institutional academic Level 1 Trauma Center. Patients between 15 and 65 years of age with a minimum of six-month follow-up were included in the study. The study excluded all patients treated with other techniques (e.g. intramedullary fixation), if there was a change in the method of fixation at the time of bone grafting or in the setting of pathologic fracture.

**Figure 1.**
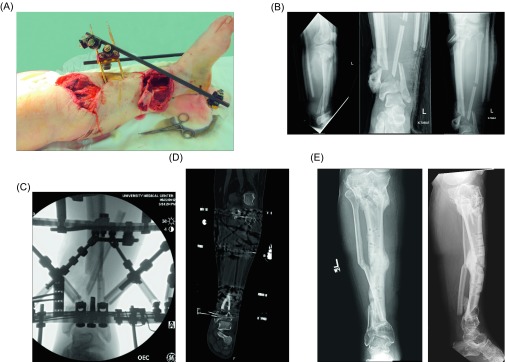
Case example of a 49-year-old male polytrauma who sustained a Gustilo-Anderson grade IIIB open left proximal and distal tibial fractures following airplane crash. (A) Clinical images of large anteromedial wounds over the proximal and distal thirds of the leg with segmental bone loss distally, (B) anteroposterior and lateral injury radiographs demonstrating the aforementioned injuries with associated fibular fracture, (C) initial post-operative fluoroscopic images with Taylor Spatial Frame (TSF) in place, (D) coronal CT renderings of the distal tibial fracture at the time of nonunion (seven months following injury) with Taylor Spatial Frame in place, and (E) final film taken one year following RIA at the time of union and TSF removal.

Fifteen patients met the inclusion/exclusion criteria. Three patients were initially stabilized with a multiplanar external fixator (either Ilizarov or Taylor Spatial Frame) and 12 provisionally with a monoplanar external fixator and subsequently converted to a circular frame during the same hospital admission. Demographic information (age, gender, comorbidities, tobacco/drug/alcohol use), injury variables (location and degree of bone loss, mechanism of injury), and surgical characteristics (Gustilo-Anderson classification, method of fixation, RIA volume, and harvest site) were extracted from the electronic medical record. The length of segmental bone loss was measured as the largest linear gap on plain film radiographs at the time of RIA. The primary outcomes of interest were presence of union, time to union, and incidence of revision surgery. Union was determined both clinically and radiographically. Clinical union was defined as pain-free full weight-bearing and radiographic union was defined as bridging callus of at least three of four cortices on final imaging. The computed tomography (CT) scan was obtained in the setting of uncertainty of radiographic union on plain film radiographs. Complications and all-cause reoperation were also recorded as a secondary endpoint. Statistical means and standard deviations were calculated for continuous variables and categorical data was expressed as frequencies (Figure 1).

### Surgical technique

Each patient underwent at least one round of aggressive debridement of the open fracture and eight required flap coverage of their injuries prior to discharge. Following discharge, patients were followed clinically, documenting weight-bearing status, symptomatology, and radiographic progression of healing. If the patient had significant bone loss (>4 cm), bone transport was performed. Nonunion was diagnosed in all cases as failure to demonstrate progressive healing on serial radiographs. Following diagnosis of nonunion, all patients subsequently returned to the operating room for RIA autografting while retaining the same method of fixation (circular fixator). Patients were not subjected to prolonged compression or compression-distraction (Accordion technique) through the nonunion site. ABG via RIA was part of the docking site surgery in patients with bone transport (bifocal treatment). Graft was preferentially harvested from the ipsilateral femur, utilizing the contralateral femur in cases in which the ipsilateral femur was instrumented or additional graft was required. The starting point was lateral entry through the proximal femur in all patients. The size of the reamer was determined using specialized radiographic ruler © (Synthes, PA) and fluoroscopy. Frame dynamization was done before frame removal. Frame removal was done as an outpatient procedure.

## Results

Of the 15 consecutive patients included in the present analysis (Table 1), the average age was 41.1 ± 14.0 (range 15–64) years and 67% (*n* = 10) were male. Two patients were diabetic and 33% (*n* = 5) reported routine tobacco use. Seven injuries were Gustilo-Anderson IIIA and eight were IIIB injuries, requiring either fasciocutaneous or myofasciocutaneous coverage. Most injuries involved the distal tibia (*n* = 8) and were metaphyseal (*n* = 8). Segmental defects ranged from 0.6 to 7.6 cm with an average linear size of 3.8 cm at the time of injury. All injuries were high-energy and sustained most often from motor vehicle (*n* = 5) and motorcycle collisions (*n* = 4). Most patients had concomitant orthopedic injuries as well (*n* = 11, 73.3%). The average time to surgery for nonunion from injury was 11.2 ± 9.5 (range 3.1–35.3) months. Three (20%) patients experienced infection. There were no infections in the donor site and all infections occurred within the soft tissues peripheral to the fracture site secondary to the soft tissue injury sustained at the time of injury. These cases were treated with debridement, irrigation, and negative pressure wound therapy, all with complete resolution of the infection. At the time of RIA (average 15 ± 12.3 months), all infections had been treated to resolution. Four patients required RIA for nonunion at the docking site following bone transport while the remaining 11 underwent RIA for nonunion of residual segmental defects as monofocal treatment. RIA was harvested from the femur in all cases, most of which were ipsilateral (*n* = 11) to the side of injury. Two patients underwent a secondary RIA harvest for persistent nonunion at four and eight months following the index RIA autografting, respectively. The average volume of RIA harvested was 34 ± 15 (range 14–60) mL. No intraoperative complications occurred as a result of RIA harvest; however, one patient experienced a femoral shaft fracture four months following RIA that required intramedullary fixation. At an average final follow-up of 13.3 ± 6.8 (6.0–31.2) months; all patients (100%) went on to union both clinically and radiographically (including the two patients who required repeat RIA). The average time to union was 6.0 ± 6.3 (1.4–25.9) months. Twelve patients (80%) went on to union within six months and 13 (86.7%) within one year. Patients were advanced to full weight-bearing by 5.3 months post-operatively on average.

**Table 1.** Patient characteristics.

No	Demographics				Injury characteristics		Surgical variables			Outcomes			
	Age	Sex	Comorbidities	Grade	Location	Defect (cm)	Infected	RIA volume (mL)	Harvest site	Union	Time to union (mo)	Complications	Reoperations
1	25	F	N	3B	Mid/Diaphyseal	4.1	N	30	Ipsi	Y	3	None	None
2	40	M	Tob	3A	Dist/Metaphyseal	3.0	N	14	Ipsi	Y	7	None	None
3	15	M	N	3B	Dist/Diaphyseal	0.6	N	30	Ipsi	Y	5	None	None
4	22	M	Tob	3B	Prox/Diaphyseal	5.0	N	19/18	Ipsi/Contra	Y	26/17	Infection; Femur fracture s/p RIA	5 (3 I&D; 1 IMN for femur fracture; 2 RIA)
5	19	M	N	3A	Mid/Diaphyseal	4.4	Y	30	Ipsi	Y	6	None	None
6	48	M	N	3B	Dist/Metaphyseal	4.0	N	20	Ipsi	Y	4	None	None
7	46	F	N	3B	Mid/Diaphyseal	1.5	Y	30	Ipsi	Y	5	Infection	1 (I&D)
8	49	M	N	3B	Prox/Metaphyseal	4.5	N	25	Contra	Y	6	None	None
9	45	F	N	3A	Dist/Metaphyseal	3.2	N	60/40	Ipsi/Ipsi	Y	2/1	Refracture of nonunion site	1 (2nd RIA)
10	64	M	DM	3B	Prox/Metaphyseal	7.6	N	35	Ipsi	Y	3	Varus deformity/shortening	None
11	48	F	DM	3A	Dist/Metaphyseal	4.5	N	40	Contra	Y	2	Infection	1 (I&D)
12	54	M	N	3A	Dist/Metaphyseal	4.4	N	35	Ipsi	Y	1.5	None	None
13	44	M	Tob	3B	Dist/Diaphyseal	5.6	N	60	Contra	Y	1	None	None
14	48	M	Tob	3A	Mid/Diaphyseal	1.9	Y	30	Ipsi	Y	13	None	None
15	43	F	Tob	3A	Dist/Metaphyseal	3.5	N	60	Ipsi	Y	2.5	None	None

M – Male; F – Female; N – No; Y – Yes; Tob – Tobacco; DM – Diabetes mellitus; Prox – Proximal; Dist – Distal; I&D – Irrigation & Debridement; IMN – Intramedullary nail; RIA – Reamer-Irrigation-Aspirator; S/p – Status post.

Five patients experienced a total of six post-operative complications including three infections (all of which required return to the operating room for formal irrigation and debridement), one patient who fell and refractured through the tibial nonunion site four months after RIA and required open reduction with internal fixation with supplemental contralateral femoral RIA, and one patient who gradually developed varus deformity and shortening through the healed nonunion site 17 months following RIA and removal of the circular fixator that was treated with a corrective tibial osteoplasty lengthening and application of a circular external fixator. Four patients underwent reoperation (for an average of two reoperations per patient), including three who required irrigation and debridement, and two who required second RIA. Although infection occurred in 20% of the patients, one patient was a smoker, another was diabetic, and one had a previously infected type IIIB open tibial fracture (see Table 1). At the time of final follow-up all patients had undergone ringed external fixator removal.

## Discussion

Nonunion following open tibial shaft fractures is not infrequent, cited as high as 12%–20% [2, 3]. The impact of nonunion is significant, with a recent study showing that reduction in quality of life was greater for long-bone nonunion than for HIV, stroke, or diabetes [11]. In the setting of high-energy injuries, tibial nonunion may be attributable to multiple factors including soft tissue compromise, resulting dysvascularity, and bone loss. A number of treatments have furthermore been proposed, each with varying success, in addressing nonunion following open tibial shaft fractures [12–14]. In this series, the study found that ABG via RIA offered a reliable solution for nonunion of open Gustilo-Anderson type III injuries of the tibia following ringed external fixator application without the need to change the method of fixation or shift to internal fixation. Despite the severity of the injuries and prevalence of comorbidities, all fractures went on to union at an average of six months following RIA. Two patients in this series required a subsequent RIA autografting procedure secondary to persistent nonunion despite initial RIA. Complications were minimal though they frequently required reoperation.

RIA has recently emerged as a promising alternative technique by which to obtain large volumes of high-quality autograft that may be used to address segmental bone loss and nonunion [5]. The histologic profile of RIA has been shown to be comparable to the gold standard of iliac crest autograft [4, 15, 16]. In addition, RIA has the added advantages of being less invasive and thereby offering reduced donor site morbidity, providing significantly larger volumes of autograft with a lower harvest time, overall cost, and a lower rate of complications [17–19]. Mesenchymal stem cells (MSCs) are a quintessential component of ABG that have been shown to accelerate fracture repair and stimulate healing of nonunions [17]. However, reliably obtaining high concentrations of MSCs is inconsistent with classic bone grafting techniques. In fact, studies analyzing the “gold standard” iliac crest aspirate have found MSCs to form only 0.001%–0.01% of cells [20, 21]. Although it is well known that MSCs can be isolated from reaming debris, RIA has been shown to be superior to classic bone graft in that MSCs can be extracted from both bony fragments as well as the filtrate bag, which is usually seen as waste [22]. In an analysis of cell cultures, Van der Bel and Blokhuis [23] found that RIA-derived cells showed a significant increase of matrix mineralization compared to iliac crest bone graft (*p* = 0.0313). They found that the osteogenic differentiation capacity of cell populations isolated from RIA ABG surpasses that of the iliac crest derived cells. The high volume, high concentration source of MSCs obtained from RIA may support our findings that RIA ABG integrates and supports local bone biology better thereby decreasing time to union. For this reason, RIA has been suggested as the new gold standard [9].

RIA has not previously been shown to serve as an alternative to compression and compression-distraction techniques through ringed external fixation. Literature evaluating the osteogenic potential and reliability of RIA for addressing bone defects [5] as well as nonunion following long-bone fractures [1, 9, 10] is sparse. In a retrospective review of 25 patients who underwent RIA for nonunion of 27 long-bone fractures with associated bone loss, Stafford et al reported that 70% of patients went on to union by six months and 90% by one year post-operatively [9]. Nineteen of these cases involved tibial nonunion, though only eight were open fractures and five patients (26%) required flap coverage. All patients in our series experienced open tibial fractures and nearly one-half (53%) required flap coverage. Furthermore, all of our patients were managed with ringed external fixator application. However, Stafford et al. stabilized their injuries with internal fixation and while the authors attempted to maintain fixation in place, conversion to temporary external fixation was often necessary in the setting of infection [9]. McCall et al. similarly reviewed 21 cases of segmental long-bone defects (of which 15 were tibial) averaging 6.6 cm that underwent RIA autografting and found that the majority (85%) went on to unite by 11 months [5]. The authors noted that tightly-packed graft did not incorporate so reliably. Our results are comparable to those mentioned in the existing literature. By one year, 13 of our 15 patients (86.7%) went on to union. The remaining two eventually united, one following multiple rounds of debridement for infected nonunion and the other following a second RIA for persistent nonunion.

Bone transport was utilized initially for four patients in this series who went on to docking site nonunion, all of which responded to RIA. RIA has not previously been described for docking site nonunion. Paley and Maar performed bone transport through an Ilizarov circular external fixator for 19 patients with segmental defects of the tibia averaging 10 cm [1]. While all cases went on to union, 10 required grafting of the docking site and the average time in the fixator was 17 months. Dendrinos et al. reported three patients of 28 who similarly required bone grafting for docking site nonunion following bone transport for open tibial fractures with bone loss [10]. Patients were maintained within the Ilizarov fixator for an average of 10 months at which time 96% of patients went on to union. All three patients who required grafting of the docking site attained union. The findings of this current study support the efficacy of RIA for nonunion in patients with open Gustilo-Anderson type III tibial fractures following multiplanar external fixator application. Similar to these series, the external fixator was maintained in all cases and did not require conversion to other means of fixation in order to attain union. While all patients eventually went on to union, two required supplemental grafting with repeat RIA due to persistent nonunion. At six months, all but three patients (80%) went on to union, and all but two did so (86.7%) within one year. Infection was uncommon (20%), with all three patients having significant comorbidities contributing to their complication; almost all cases required return to the operating room for formal debridement. Therefore, patients should be counseled on the high likelihood of eventual union but with the moderate expectation of reoperation (26.7%).

The average time to union following RIA bone grafting was six months in our series. While the success rate was considerable, the duration of treatment and time within the frame should be communicated to the patients and their families in order to mitigate expectations. Furthermore, despite the growing evidence in favor of RIA ABG, patients should be counseled on the associated complications unique to this technique including fracture through the long-bone harvest site.

Our investigation was primarily limited by its power, retrospective nature, and short-term follow-up. However, all patients were followed until both clinical and radiographic union. These findings contribute to the growing body of evidence supporting ABG via RIA as an appealing new standard for addressing difficult nonunions, both in the setting of segmental defects as well as docking site nonunion following bone transport.

## Conclusion

The current study shows that ABG via RIA offers a reliable solution to nonunion of open Gustilo-Anderson type III tibial fractures treated with ringed external fixator application. The same method of ring external fixation can furthermore be maintained with the addition of bone grafting. Furthermore, RIA may be utilized in treating docking site nonunion following bone transport. The high volume and concentration of MSCs in RIA ABG foreseeably contributes to the success of this procedure. All 15 patients in our series reliably went on to union at an average of six months. Complications were minimal, but infection occurred in 20% of cases and required formal debridement in all cases. Larger comparative clinical investigations are warranted to determine the relative efficacy of RIA over conventional ABG for complex tibial nonunion treated with Taylor Spatial Frame (TSF).

## Conflict of interest

The authors declare that there is no conflict of interest regarding the publication of this paper.
